# Zwangsmedikation psychisch erkrankter Menschen im Justizvollzug: Situation in Deutschland und Empfehlungen für die Praxis

**DOI:** 10.1007/s00115-020-00957-2

**Published:** 2020-07-17

**Authors:** Johannes Fuß, Inga Marquardt, Peer Briken, Norbert Konrad

**Affiliations:** 1 Institut für Sexualforschung, Sexualmedizin und Forensische Psychiatrie, Zentrum für Psychosoziale Medizin, Universitätsklinikum Hamburg-Eppendorf, Martinistr. 52, 20246 Hamburg, Deutschland; 2 Amtsgericht Hamburg, Hamburg, Deutschland; 3 Institut für Forensische Psychiatrie, Charité – Universitätsmedizin Berlin, Berlin, Deutschland

**Keywords:** Gefängnispsychiatrie, Strafgefangene, Zwangsmaßnahmen, Zwangsbehandlung, Medizinethik, Prison psychiatry, Inmates, Compulsory measures, Compulsory treatment, Medical ethics

## Abstract

**Hintergrund:**

Medizinische Zwangsmaßnahmen stellen stets einen Eingriff in das Grundrecht auf körperliche Unversehrtheit sowie das Recht auf Selbstbestimmung des Patienten dar. Ist ein Patient inhaftiert, mithin ohnehin bereits in seinen Grundrechten stark eingeschränkt, und muss die Zwangsmaßnahme zudem in einer Justizvollzugsanstalt stattfinden, so stellt dies für alle Beteiligten – insbesondere für den anordnenden Psychiater – eine besondere Herausforderung dar.

**Ziel und Methode:**

Dieser Artikel soll die psychiatrische Versorgung psychisch erkrankter Menschen im Justizvollzug darstellen, die juristischen Voraussetzungen einer Zwangsmedikation erläutern und Empfehlungen für geeignete Rahmenbedingungen geben.

**Ergebnisse und Diskussion:**

Für psychisch erkrankte Menschen in Haft gibt es in Deutschland keine Pflichtversorgungslösung entsprechend der Psychisch-Kranken-Gesetze, sodass diese bei schweren Erkrankungen nicht regelhaft einer stationär-psychiatrischen Versorgung zugeführt werden können. Dies kann zur Folge haben, dass bei ungünstigen Krankheitsverläufen eine Zwangsmedikation innerhalb des Justizvollzugs erwogen werden muss. Die Zwangsmedikation im deutschen Justizvollzug nimmt im Vergleich zur Zwangsmedikation in psychiatrischen Kliniken dabei eine Sonderstellung ein, da Ärzte in den meisten Bundesländern eine solche ohne richterliche Beteiligung anordnen können. Die Landesgesetzgeber sollten prüfen, ob hier eine gesetzliche Anpassung erforderlich oder sinnvoll wäre. Da die Umstände (rechtlich und versorgungstechnisch) für inhaftierte Patienten von denen in Kliniken stark abweichen, sollten die Rahmenbedingungen einer Zwangsmedikation wohl überlegt sein. Wir empfehlen das Hinzuziehen einer ethischen Fallberatung, wenn die Entscheidung ohne richterliche Beteiligung getroffen wird, sowie die Verlegung auf psychiatrische oder zumindest medizinische Krankenhausstationen zur Durchführung der Zwangsmedikation.

## Abstract (en)

**Background:**

Coercive medical measures always represent an encroachment on the patient’s basic right to physical integrity and right to self-determination. If a patient is imprisoned, his basic rights are already severely restricted, and the coercive measure must also take place in a correctional facility. This amounts to a special challenge for everyone involved—but especially for the psychiatrist in charge.

**Objectives and Methods:**

This article is aimed at describing the psychiatric care of people with mental disorders in prisons, explaining the legal requirements for compulsory medication and giving recommendations for suitable conditions.

**Results and Conclusions:**

In Germany, there is no legally regulated health-care solution for people with mental disorders in custody in accordance with the laws on the mentally ill of the German federal states (Psychisch-Kranken-Gesetze). Therefore, in the case of serious mental disorders, imprisoned patients cannot be regularly provided with inpatient psychiatric care. As a result, compulsory medication within the correctional system must be considered if the course of the mental disorder is unfavorable. Compulsory medication in the German prison system has a special position compared with compulsory medication in psychiatric clinics, as doctors in most federal states can prescribe such medication without judicial involvement. The state legislators should check whether a legal adjustment would be necessary or helpful here. As the circumstances (legal and care-related) for imprisoned patients differ greatly from those of patients in clinics, the general conditions for compulsory medication should be carefully considered. We recommend consulting ethical case counseling if the decision is made without judicial involvement, and moving imprisoned patients to a psychiatric or at least to a medical ward before the administration of compulsory medication.

## Hintergrund

In Westeuropa und den USA wird seit über 80 Jahren über einen Zusammenhang zwischen der Zahl stationär-psychiatrischer Betten und der Zahl an Gefängnisinsassen diskutiert. Wenn psychiatrische Betten abgebaut werden, komme – so die als Penrose-Hypothese bekannt gewordene Theorie [1] – ein Teil der zuvor stationär behandelten Menschen früher oder später in Gefängnissen oder forensisch-psychiatrischen Einrichtungen unter. Die Theorie wurde immer wieder kritisiert und relativiert, jedoch untermauern mehrere longitudinale Studien [2–4], dass der zunehmende Abbau allgemeinpsychiatrischer Betten und die Verkürzung der Liegezeiten im Zuge der Deinstitutionalisierung mit einer drastischen Zunahme der Betten in forensisch-psychiatrischen Krankenhäusern und Gefängnissen einherging.^1^ Eine Schlussfolgerung aus der Penrose-Hypothese könnte sein, dass ein Teil der psychisch erkrankten Menschen ein stark strukturiertes Setting benötigen, das ihre Medikamenteneinnahme sicherstellt und Kontrollfunktionen übernimmt. Für Deutschland ist diese Entwicklung erst seit Mitte der 1990er-Jahre festzustellen [5]. Die angenommene Verlagerung schwer erkrankter Patienten von stationären Allgemeinpsychiatrien in Gefängnisse [6] führte zu kritischen Leitartikeln wie „Bring back the asylum“ [7], „Does deinstitutionalization cause criminalization“ [8]? oder „Prisons as new asylums“ [9]. Gegenpositionen (z. B. [10]) postulieren, dass auch voneinander unabhängige Entwicklungen die gegenläufigen Belegungstrends in der Allgemeinpsychiatrie und forensischen Psychiatrie bzw. im Justizvollzug erklären können. Einigkeit dürfte aber darüber bestehen, dass psychische Erkrankungen in Gefängnissen schlechter behandelt werden können als in psychiatrischen Einrichtungen. Trotzdem kommen psychische Erkrankungen bei Menschen im Justizvollzug häufig vor [11–13]. Möglicherweise hat die Zahl schwer erkrankter Patienten, die zusätzlich oft obdachlos, alleinstehend und arbeitslos sind [14], in deutschen Gefängnissen zugenommen. In der Berliner Gefängnispsychiatrie waren Ende Januar 2020 über 80 % der Patienten obdachlos, gegenüber ca. 13 % der Patienten in allgemeinpsychiatrischen Versorgungskliniken [15]. Obwohl Längsschnittstudien für Deutschland fehlen, kann bei aktuell circa 50.000 inhaftierten Strafgefangenen [16] und circa 15.000 Untersuchungshaftgefangenen [17] davon ausgegangen werden, dass mindestens 2500 Menschen in Haft unter Psychosen leiden – die Zahl an Menschen mit affektiven Erkrankungen dürfte deutlich höher liegen. Diese „Forensifizierung“ schwer kranker Patienten dürfte auch begünstigt haben, dass die Rechtsprechung die zu begründenden Voraussetzungen für eine Zwangsmedikation außerhalb des Justizvollzuges in den letzten Jahren konkretisiert und dadurch erhöht hat. Dies dürfte eine Abnahme der Zwangsmedikationen zur Folge gehabt haben [18].^2^ Ein Zusammenhang zwischen unterlassener Zwangsmedikation und der Zunahme aggressiver Übergriffe ist wissenschaftlich gut belegt [19]. Dies lässt die Vermutung zu, dass einzelne psychisch erkrankte Menschen aufgrund einer unterlassenen Zwangsmedikation straffällig geworden sind und so in den Justizvollzug gelangten. Auch in Zukunft ist daher zu befürchten, dass bei gleichbleibenden Rahmenbedingungen die Zahl der inhaftierten Menschen, die nicht behandelt, schwer krank und weder krankheits- noch behandlungseinsichtig sind, hoch bleiben wird. Bei schweren Verhaltensstörungen mit Eigen- und Fremdgefährdung wird sich immer wieder die Frage einer Zwangsmedikation in Haft stellen. Zahlen zur Häufigkeit von psychiatrischen Zwangsmedikationen in Haft liegen jedoch ebenso wenig wie Leitlinien oder Empfehlungen für ihre Durchführung vor. Der vorliegende Artikel soll deshalb nach einer Einführung zur Versorgungssituation psychisch erkrankter Menschen in deutschen Gefängnissen auf die juristischen Voraussetzungen und geeignete Rahmenbedingungen für Zwangsmedikation in Haft eingehen.

## Die Versorgung psychisch erkrankter Menschen in Haft

Ein großer Teil der psychisch erkrankten Menschen in Haft wird mithilfe von konsiliarisch tätigen Fachärzten für Psychiatrie und Psychotherapie in den Anstalten ambulant behandelt [5]. Diese psychiatrische Grundversorgung kann aufgrund eines Mangels an Psychiatern vor allem in Anstalten der ländlichen Regionen oft nicht ausreichend gewährleistet werden und es wird daher aktuell geprüft, ob in Zukunft telemedizinische Modellprojekte die langfristige ambulante Versorgung ergänzen können [20].

Wenn psychische Erkrankungen so schwer ausgeprägt sind, dass eine stationäre Behandlung notwendig wäre, fehlt für Menschen in Haft eine einheitliche Lösung. Anders als außerhalb der Gefängnisse, wo durch die regionale Pflichtversorgung psychisch erkrankte Menschen in Deutschland ein zeitnahes (bei Notfällen auch unmittelbares) stationäres Behandlungsangebot erhalten, gibt es für psychisch erkrankte Menschen in Haft keine Pflichtversorgungslösung [21]. Einige Länder (Baden-Württemberg, Bayern, Berlin, Brandenburg, Hessen, Niedersachsen, Nordrhein-Westfalen, Rheinland-Pfalz, Sachsen und Schleswig-Holstein) verfügen zwar über psychiatrische Abteilungen innerhalb des Justizvollzuges, allerdings gilt hier keine Aufnahmepflicht, sodass einerseits Wartelisten bestehen und andererseits keine stationäre Aufnahme erfolgen kann, wenn die Abteilungen voll belegt sind. Ein Vorhalten von Kapazitätsreserven für ungeplante Aufnahmen, wie in der regionalen Pflichtversorgung, findet in psychiatrischen Abteilungen des Justizvollzuges nicht statt. Andere Länder (z. B. Hamburg und Mecklenburg-Vorpommern) verlegen psychisch erkrankte Menschen aus der Haft in eine Maßregelvollzugsklinik, wenn eine stationäre Behandlung notwendig wird, da diese Kliniken über hohe Sicherheitsstandards und die Möglichkeiten einer leitliniengerechten Behandlung verfügen. Hierfür sind allerdings freie Kapazitäten im Maßregelvollzug notwendig, da die Übernahme von psychisch erkrankten Strafgefangenen nicht deren primärer Versorgungsauftrag ist, die aber nicht immer gegeben sind. Angesichts der Zunahme psychiatrischer Unterbringungen im Maßregelvollzug in den letzten Jahren [22] dürfte dieser Weg mancherorts komplizierter geworden sein. Dies kann dazu führen, dass lange Wartezeiten entstehen, bis eine störungsspezifische stationäre Behandlung begonnen werden kann. Eine weitere Möglichkeit zur leitliniengerechten Behandlung psychisch erkrankter Menschen ist die psychiatrische Behandlung in allgemeinpsychiatrischen Einrichtungen außerhalb des Justizvollzugs. Dies wird jedoch selten umgesetzt [23], was verschiedene Gründe hat, wie z. B. die als ungünstig angesehene Präsenz von Justizvollzugsbeamten auf allgemeinpsychiatrischen Stationen, die dort mindestens zu zweit für die Bewachung der Strafgefangenen während der Behandlung zuständig sind. Dass die Anwesenheit von Justizvollzugsbeamten auf akutpsychiatrischen Stationen bei psychotischen Patienten Ängste auslösen und deren Krankheitsverlauf beeinflussen kann, liegt nahe. Die letzte Möglichkeit, um eine leitliniengerechte Behandlung psychisch erkrankter Menschen in Haft zu ermöglichen, bietet die Haftunterbrechung gem. § 455 Strafprozessordnung (StPO), über die die zuständige Strafvollstreckungsbehörde entscheidet. Neben der Feststellung einer gesundheitsbedingten Vollzugsuntauglichkeit dürfen insbesondere auch Gründe der öffentlichen Sicherheit nicht entgegenstehen, was in der Praxis diesen Weg häufig versperrt [24]. Da als Rechtsfolge die bereits in Vollstreckung befindliche Strafhaft unterbrochen wird, stellt dieses Verfahren jedoch formal keine Behandlung von Strafgefangenen dar.

Diese Zusammenfassung der Versorgungsmöglichkeiten verdeutlicht, dass in weiten Teilen der Bundesrepublik psychisch erkrankten Menschen in Haft nicht verlässlich und zeitnah eine leitliniengerechte Therapie zuteilwird. Insbesondere bei schwer erkrankten Menschen, die aufgrund fehlender Krankheits- und Behandlungseinsicht eine ambulant empfohlene Therapie mit Psychopharmaka ablehnen, kann dies zu äußerst ungünstigen Verläufen führen, die mit Eigen- und Fremdgefährdung einhergehen. Vonseiten der Anstalten erfolgt dann in der Regel – wenn keine Verlegung in eine psychiatrische Abteilung möglich ist – eine Verlegung auf sogenannte Beobachtungs- und/oder besonders gesicherte Hafträume, die faktisch zu einer Isolation der Menschen führt. Manche dieser Räume sind mit Videokameras ausgestattet, sodass die Patienten überwacht werden können, was die Privatsphäre stark einschränkt. Vor allem Menschen mit psychotischen Störungen, Selbstverletzungen und Suizidalität werden so untergebracht und verbleiben mangels Alternativen und anhaltenden Gefährdungsaspekten gegebenenfalls für Monate in diesen Hafträumen. Für gewöhnlich führt dies zu einer Verschlechterung von psychopathologischen Symptomen, sodass es im Zeitverlauf vereinzelt zu schwergradigen Zustandsbildern mit Verwahrlosung, Denkzerfahrenheit und ausgeprägten inhaltlichen Denkstörungen der Patienten kommen kann. Eine Einwilligung zur Medikamenteneinnahme ist unter diesen Umständen meist nur noch schwer zu erreichen und auch die Möglichkeiten für psychotherapeutische und psychosoziale Interventionen sind stark eingeschränkt, da vor allem kontinuierliche Pflege durch ein multiprofessionelles Team fehlt. Dass diese Versorgung schwer erkrankter Patienten nicht dem normierten Äquivalenzprinzip^3^ – nach dem die medizinischen Leistungen im vollzuglichen Gesundheitswesen den Leistungen für gesetzlich Krankenversicherte gleichwertig sein müssen – entspricht, ist kaum zu bestreiten. Darüber hinaus können diese Zustände im Hinblick auf das Recht auf körperliche Unversehrtheit (neben einer Gefährdung für die Gesundheit der Patienten und bei bestehender Fremdgefährdung auch für das Personal) sowie im Hinblick auf eine menschenwürdige Unterbringung (bei andauernder Isolation) hochproblematisch sein. Außerdem werden die konsiliarisch tätigen Psychiater in eine Situation versetzt, in der sie sich aufgrund des dargestellten strukturellen Versorgungsproblems bei Nichthandeln jedenfalls fragen müssen, ob der Tatbestand einer unterlassene Hilfeleistung im Raum steht. An Handlungsoption bleibt ihnen jedoch häufig nur noch eine – ebenfalls stark in die Rechte des Patienten eingreifende – Medikation gegen dessen Willen.

## Die juristischen Voraussetzungen für Zwangsmedikation in Haft

Gemäß § 101 Strafvollzugsgesetz (StVollzG) sind Zwangsmaßnahmen auf dem Gebiet der Gesundheitsfürsorge nur bei Gefahr für das Leben oder schwerwiegender Gefahr für die Gesundheit der Gefangenen zulässig, wenn diese krankheitsbedingt zur Einsicht in die Notwendigkeit der Behandlungsmaßnahmen nicht fähig sind, oder bei Gefahr für die Gesundheit Dritter. Bei Letzterem dürfte der Gesetzgeber eher an ansteckende Krankheiten und an eine dadurch bedingte Gefährdung von Besuchern, Bediensteten und/oder Mitgefangenen gedacht haben. Im Fall der psychiatrischen Erkrankung kann das Tatbestandsmerkmal der Gefährdung Dritter jedoch (ergänzend) eine Rolle spielen, wenn der psychisch erkrankte Mensch aggressives Verhalten an den Tag legt. Die Maßnahmen müssen nach der bundesgesetzlichen Regelung darüber hinaus für alle Beteiligten zumutbar sein, woran es beispielsweise fehlen kann, wenn ein Eingriff die Menschenwürde verletzt.^4^ Der Schwerpunkt dürfte in der Begründung der Verhältnismäßigkeit liegen: Die Maßnahme muss geeignet, erforderlich und im engeren Sinne angemessen sein, um als rechtmäßig zu gelten, und darf gemäß § 101 Absatz 3 StVollzG nur auf Anordnung und unter Leitung eines Arztes durchgeführt werden, außer es liegt eine lebensgefährliche Situation vor, die die Beteiligung eines Arztes nicht (mehr) zulässt.

Die Bundesländer haben eigene Regelungen getroffen, die gegenüber § 101 StVollzG teilweise weitergehende und differenzierte Voraussetzungen festlegen, was die Subsumtion im hier zu betrachtenden Fall der psychischen Erkrankung jedoch nicht einfacher macht. So dürften beispielsweise bestimmte Fälle der Zwangsernährung – jedenfalls von außen betrachtet – einfacher nachzuvollziehen sein, wenn Lebensgefahr besteht: Unterbleibt sie, droht der Tod des Gefangenen und mildere Mittel dürften nach Ablauf einer gewissen Zeit kaum ersichtlich sein. Bei einer psychischen Erkrankung macht bereits die Begründung einer Gesundheitsgefährdung Schwierigkeiten: Nach der kommentierenden Literatur zu § 101 StVollzG setzt die schwerwiegende Gefahr für die Gesundheit voraus, dass eine dauerhafte oder zumindest lang andauernde Beeinträchtigung wichtiger Körperfunktionen droht.^5^ Diese Definition ist auf psychopathologische Befunde nicht zugeschnitten. In Anlehnung an Zwangsbehandlungen nach Bürgerlichem Gesetzbuch (vgl. § 1906a BGB) wird mithin zu begründen sein, dass gewichtige gesundheitliche Nachteile mit der Maßnahme abgewendet werden können, die nicht zwingend organischer Natur sein müssen und bspw. in der Verhinderung einer Chronifizierung des Krankheitsbildes liegen können. Ist eine Chronifizierung bereits eingetreten, muss eine dem Patienten schadende erhebliche Verschlechterung des Gesundheitszustandes aufgrund weiterer Umstände begründet werden. Ergänzend wird sich in der Praxis der Psychiater auf die Gefährdung Dritter stützen können, soweit sich die psychische Erkrankung auch in aggressivem Verhalten äußert. Für sich alleine stehen kann dieses Tatbestandsmerkmal jedoch nicht, da dieser Gefährdung auch durch Isolation des psychisch erkrankten Menschen in besonders gesicherten oder Beobachtungshafträumen begegnet werden kann, mithin ein (vermeintlich) milderes Mittel zur Verfügung steht. In der Praxis ist die Isolation in der Regel ohnehin das zunächst gewählte Mittel, und dies grundsätzlich auch im Sinne der Verhältnismäßigkeit: Durch die Separierung und Beobachtung des psychisch erkrankten Menschen lässt sich das Risiko für Eigen- und Fremdgefährdung zunächst senken. Auch der Gesundheitszustand kann sich durch eine reizarme Umgebung in manchen Fällen verbessern. Diese Art der Unterbringung ist eine vollzugliche Maßnahme, keine medizinische, und erfolgt „lediglich“ unter Beteiligung eines Arztes. Dies gilt (außer in Baden-Württemberg) auch für die Fixierung, die ebenso – auf Zeit – die Selbstverletzung und Gefährdung Dritter ausschließen kann, aber selten zur Verbesserung des Gesundheitszustandes beitragen dürfte. Dauert eine isolierte Unterbringung über Wochen oder Monate ohne ersichtliche Zustandsverbesserung an, stellt sich folglich erneut die Frage der Behandlung: Der Psychiater wird dabei vor die Aufgabe gestellt, nicht nur die Alternativlosigkeit (kein milderes Mittel), sondern auch den Erfolg der Behandlung vorauszusagen (Eignung). Beispielhaft kann dies beim Vorliegen einer akuten psychotischen Episode im Rahmen einer paranoiden Schizophrenie anhand der Studienlage und klinischer Erfahrungswerte erfolgen. Die zudem erforderliche Feststellung der fehlenden Einsichtsfähigkeit im Hinblick auf den Behandlungsbedarf dürfte sich in der Praxis beim Vorliegen schwerer psychischer Erkrankungen mit ausgeprägten Denkstörungen im Einzelfall aus dem psychopathologischen Befund ergeben. Auch sie ist aber eine erforderliche Voraussetzung und zu bejahen, sofern der psychisch erkrankte Mensch zur Einsicht in die Schwere seiner Krankheit und die Notwendigkeit von Behandlungsmaßnahmen oder zum Handeln gemäß solcher Einsicht krankheitsbedingt nicht fähig ist.^6^

Während deutschlandweit ein Richtervorbehalt die Entscheidung über Zwangsmedikation nach Maßregelvollzugs- und Psychisch-Kranken-Gesetzen in die Hände eines Gerichts legt, kann es im Vollzug dazu kommen, dass der Psychiater alleine über die Zwangsmedikation entscheidet, teilweise mit Zustimmung der Anstaltsleitung und/oder nach Einholung einer zusätzlichen ärztlichen Begutachtung. Nur in wenigen Bundesländern entscheidet (nach ärztlicher Stellungnahme) die Anstaltsleitung selbst und lediglich in Schleswig-Holstein und Baden-Württemberg erfolgt eine „echte“ Beteiligung des Gerichts.^7^ In den übrigen Ländern wird der Gefangene über die Möglichkeit, vorab eine gerichtliche Entscheidung herbeizuführen, aufgeklärt, und es wird eine angemessene Frist vor dem Vollzug der Maßnahme abgewartet, damit diese Möglichkeit genutzt werden kann. Nachdem die Regelungen der Fixierungen im Justizvollzug eine Überarbeitung erfahren haben und eine gerichtliche Anordnung vorsehen, ist dieses Verhältnis fraglich. Da beide Vorschriften den Regelungen des unmittelbaren Zwanges und nicht denen der Gesundheitsfürsorge unterfallen, sollten die Landesgesetzgeber prüfen, ob eine Harmonisierung erforderlich, wenigstens aber sinnvoll ist. Die richterliche Entscheidung ist nach dem Bundesverfassungsgericht eine der Möglichkeiten, die der Ersetzung der fehlenden Zustimmung des Betroffenen dient. Ob mit oder ohne vorherige gerichtliche Beteiligung ist in allen Bundesländern die Maßnahme nachträglich gerichtlich überprüfbar, sodass eine Dokumentation zwingend erforderlich ist. Hilfreich kann es dabei sein, wenn Vollzug und Medizin unter Berücksichtigung datenschutzrechtlicher Belange und der ärztlichen Schweigepflicht gemeinsame Vordrucke erarbeiten, da vollzugliche und medizinische Anordnungen im Fall der Zwangsmedikation stets miteinander einhergehen. Dies gilt bereits dann, wenn vorher oder hinterher die Unterbringung in einem gesicherten (Beobachtungs‑)Haftraum oder ggf. sogar eine Fixierung zwecks Durchführung der Maßnahme erforderlich ist. Die folgenden Empfehlungen zur Handhabung, wie die Beteiligung einer klinischen Ethikkommission, können dabei ebenso Teil einer strukturierten Dokumentation sein.

## Geeignete Rahmenbedingungen für Zwangsmedikation in Haft am Beispiel der Situation in Hamburg

Im Hamburger Justizvollzug dürften Zwangsmedikationen in den letzten Jahren kaum eine praktische Bedeutung gehabt haben, da bisher schwer kranke Patienten in die hiesige forensisch-psychiatrische Klinik verlegt werden konnten^8^, bis eine deutliche Zustandsbesserung oder Therapieadhärenz erreicht wurde. Anfang 2020 sprach diese Klinik einen Aufnahmestopp aus, da es zu einer deutlichen Zunahme von Unterbringungen nach § 126a StPO gekommen war und keine Aufnahmekapazität mehr bestand [25]. Durch die veränderte Versorgungslage kam es zu einer Zwangsmedikation bei einem inhaftierten Patienten mit paranoider Schizophrenie. Neben den oben beschriebenen juristischen Voraussetzungen stellte sich auch die Frage einer ethischen Abwägung und eines bestmöglichen Vorgehens. Als Ergebnis dieses Prozesses wurden die folgenden Empfehlungen für Zwangsmedikationen im Justizvollzug entwickelt:

### Abwägung des ethischen Dilemmas und Überprüfung der Indikation

Die Zwangsmedikation von Menschen, deren Grundrechte bereits durch die Inhaftierung eingeschränkt sind, ist ein schwieriges medizinethisches Thema und gesellschaftlich besonders umstritten. Wenn eine psychiatrische Zwangsmedikation nicht auf einer psychiatrischen Krankenhausstation mit entsprechend geschultem Personal erfolgt, stellt dies ein Risiko dar. Zum Beispiel kann eine Zwangsmedikation im Vollzug traumatisierender als in einem Krankenhaus erlebt werden, da die Prozedere weniger auf die Bedürfnisse von psychiatrischen Patienten abgestimmt sind. Die Behandler befinden sich in einer klassischen Dilemmasituation, in der sie zwischen zwei ungünstigen Szenarien wählen müssen (Zwangsmedikation im Vollzug vs. Nichthandeln), weil das erwünschte Szenario (Verlegung auf eine psychiatrische Station zur Durchführung der Zwangsmedikation) aufgrund unzureichender Versorgungsbedingungen häufig nicht möglich ist. Obwohl die Behandler die Versorgungsbedingungen in der Regel kaum beeinflussen können, müssen sie das ärztliche Vorgehen unter diesen Bedingungen verantworten. Die ausführliche ethische Abwägung einer Zwangsmedikation erfolgt außerhalb des Justizvollzugs im Rahmen der Begutachtung und der richterlichen Entscheidung. In den Bundesländern, in denen keine gerichtliche Beteiligung bei Zwangsmedikation im Justizvollzug erfolgt, sollte diese ethische Abwägung anders organisiert werden. Ähnlich wie bei anderen weitreichenden medizinischen Entscheidungen, empfiehlt sich in diesem Fall eine ethische Fallberatung, um die möglichen Szenarien abzuwägen. In den Hamburger Anstalten, die vom Universitätsklinikum Hamburg-Eppendorf psychiatrisch versorgt werden, tritt eine interdisziplinäre klinische Ethikkommission zusammen, die den Fall erörtert und ethisch aus verschiedenen Blickwinkeln beleuchtet. Außerdem muss – jedenfalls in Hamburg – eine ärztliche Zweitsicht erfolgen, die juristisch notwendig, aber auch für die indizierende Person entlastend sein kann. Dies gilt auch für die in Hamburg erforderliche Zustimmung der Anstaltsleitung. Für konsiliarisch tätige Psychiater in Gefängnissen, die nicht im Umfeld einer größeren Klinik arbeiten, sollten vergleichbare Strukturen geschaffen werden, in denen die wichtigsten Positionen und Argumente im Vorfeld einer geplanten Zwangsmedikation von einem möglichst diversen Expertengremium (z. B. Ärzte, Ethiker, Juristen, Seelsorger, Sozialarbeiter u. a.) erörtert werden können. Hier kann auch die komplizierte Rolle der Konsiliarpsychiater reflektiert werden, die sich in einem Loyalitätskonflikt zwischen Patient und Anstalt befinden können.

### Schaffung geeigneter Umgebungsbedingungen

Beobachtungs- und besonders gesicherte Hafträume einer Anstalt sind keine geeignete Umgebungsbedingung für eine Zwangsmedikation von psychisch erkrankten Menschen. Die Vermeidung einer traumatisierenden Erfahrung muss auch in Hinblick auf zukünftige Compliance bei der Durchführung hohe Priorität haben – die Behandler müssen den Prozess steuern können. Bei einer Durchführung auf einer Station des Vollzugs wäre zu befürchten, dass z. B. bei körperlicher Auseinandersetzung ein sogenannter HSP(Helm-Schild-Pfefferspray)-Einsatz zur Sicherung des Patienten und anschließender Fixierung durch den Vollzug angeordnet und vollzogen wird. Dieser Ablauf kann jedoch so schwer traumatisierend sein, dass er die positiven Effekte einer Medikation überwiegen würde. Ein solche Form der Eskalation muss unbedingt verhindert werden und sollte im Vorfeld offen mit den Akteuren besprochen werden – bei drohender Eskalation kann die Zwangsmedikation auch abgebrochen und zu einem späteren Zeitpunkt mit günstigeren Bedingungen erneut vollzogen werden. Wir empfehlen daher generell die Verlegung von Patienten auf psychiatrische, zumindest medizinische Krankenhausstationen innerhalb des Justizvollzugs. Diese sind aus ärztlicher Perspektive besser geeignet, da dort auch seltene Nebenwirkungen, wie z. B. Anaphylaxie bei erstmaliger Gabe eines Depotpräparats frühzeitig diagnostiziert und behandelt werden können. Außerdem ist es gegenüber den Patienten ein wichtiges Signal, dass die Maßnahme im klinischen Kontext stattfindet, um zu markieren, dass es sich um eine ärztliche Maßnahme (nicht um Strafe o. Ä.) handelt, auch wenn diese juristisch den Regelungen des unmittelbaren Zwanges und nicht denen der Gesundheitsfürsorge zuzuordnen ist. Es ist wichtig, das Personal dieser Stationen frühzeitig in den Prozess einzubeziehen. In Hamburg soll dies z. B. bereits durch einen teilnehmenden Vertreter im Rahmen der klinischen Ethikkommission erfolgen. Es sollte ferner geprüft werden, ob zur Durchführung einer Zwangsmedikation auf eine Fixierung, die eine hohe Belastung darstellt, verzichtet werden kann. Ein kurzes Festhalten der Patienten kann genügen, um eine Depotmedikation zu verabreichen. Wenn eine Zwangsmedikation im Vollzug angeordnet wird, sind die Patienten häufig bereits seit längerer Zeit in einem Beobachtungs- oder besonders gesicherten Haftraum untergebracht. Es kann Vollzugsbeamte geben, die sich im Rahmen der Unterbringung um ein besonders vertrauensvolles und gutes Verhältnis zu den psychisch erkrankten Menschen bemühen (vergleichbar mit einer Art Bezugspflege in der Psychiatrie). Es kann hilfreich sein, wenn diese die Maßnahme begleiten. Auch diesbezüglich sind ein enger Austausch und ein Konsens mit den Beteiligten der Anstalt erforderlich und empfehlenswert. Darüber hinaus sollten die Empfehlungen der aktuellen S3-Leitlinie „Verhinderung von Zwang: Prävention und Therapie aggressiven Verhaltens bei Erwachsenen“ beachtet werden. In Hamburg konnte durch diese Maßnahmen (insbesondere die Verlegung des Patienten in das Vollzugskrankenhaus und die Hinzuziehung von psychiatrisch versiertem Vollzugspersonal) letztlich eine drohende Eskalation verhindert werden, sodass die erfolgte Zwangsmedikation des Patienten nach Abklingen der Symptome von ihm als hilfreich eingeschätzt wurde.

## Fazit für die Praxis

- Indikationen einer Zwangsmedikation unter Haftbedingungen gehören zu den Herausforderungen an die Psychiatrie und Medizinethik.
- Anders als außerhalb des Justizvollzugs, wo ein Richtervorbehalt besteht, wird bei Zwangsmedikation von inhaftierten Menschen in den meisten Bundesländern die Entscheidung von einem Arzt (nach vorheriger Beteiligung der Anstaltsleitung oder eines weiteren Arztes) getroffen. Die Landesgesetzgeber sollten prüfen, ob hier eine gesetzliche Anpassung erforderlich oder sinnvoll wäre.
- Die Verhaltensspielräume und Grundrechte inhaftierter Patienten sind von vornherein eingeschränkt. Die Rahmenbedingungen einer Zwangsmedikation sollten daher wohlüberlegt und mit der Anstalt abgesprochen sein.
- Der zunehmende Abbau allgemeinpsychiatrischer Betten und die Verkürzung der Liegezeiten haben möglicherweise dazu beigetragen, dass einzelne psychisch erkrankte Menschen nicht behandelt, obdachlos und kriminell werden [26]. Daher wird das Thema einer Zwangsmedikation in Haft auch zukünftig eine hohe Relevanz haben.

## Footnotes

1 Diese Entwicklung wurde auch als Reinstitutionalisierung oder Transinstitutionalisierung bezeichnet.

2 Dies bezieht sich vor allem auf die Entscheidungen des Bundesverfassungsgerichts in den Jahren 2011 (BVerfGE 128, 282 ff.; BVerfGE 129, 269 ff.) und 2013 (BVerfGE 133, 112 ff.).

3 Das auf Bundesebene in § 61 StVollzG normierte Äquivalenzprinzip hat auch in den Landesvollzugsgesetzen seinen Niederschlag gefunden.

4 vgl. BeckOK Strafvollzug Bund/Wachs, 17. Ed. 01.02.2018, StVollzG § 101 Rn. 4.

5 vgl. BeckOK Strafvollzug Bund/Wachs, 17. Ed. 01.02.2018, StVollzG § 101 Rn. 3.

6 BVerfGE 128, 282 (304 f.).

7 Gemäß § 80 Abs. 3 BWJVollzGB ist eine solche Maßnahme auf Antrag der JVA nur mit vorheriger richterlicher Entscheidung des Amtsgerichts zulässig; gemäß § 86 Abs. 4 Ziffer 3 LStVollzG SH setzt eine ärztliche Zwangsmaßnahme voraus, dass das Gericht der Durchführung der Maßnahme nach Anhörung des Gefangenen zugestimmt hat.

8 Entsprechend § 63 Hamburgisches Strafvollzugsgesetz erfolgte die Verlegung zum Zweck der Behandlung.

## References

[1] Penrose LS (1939). Mental disease and crime: outline of a comparative study of European statistics. Br J Med Psychol.

[2] Mundt AP (2015). Psychiatric hospital beds and prison populations in South America since 1990: Does the Penrose hypothesis apply?. JAMA.

[3] Chow WS, Priebe S (2016). How has the extent of institutional mental healthcare changed in Western Europe? Analysis of data since 1990. BMJ Open.

[4] Priebe S (2005). Reinstitutionalisation in mental health care: comparison of data on service provision from six European countries. BMJ.

[5] Konrad N (2007). Psychiatrische Konsiliartätigkeit im deutschen Justizvollzug. Psychosom Konsiliarpsychiatr.

[6] Lamb HR, Weinberger LE (2005). The shift of psychiatric inpatient care from hospitals to jails and prisons. J. Am. Acad. Psychiatry Law.

[7] Sisti DA, Segal AG, Emanuel EJ (2015). Improving long-term psychiatric care: bring back the asylum. JAMA.

[8] Lamb HR (2015). Does deinstitutionalization cause criminalization? The penrose hypothesis. JAMA.

[9] Konrad N (2002). Prisons as new asylums. Curr Opin Psychiatry.

[10] Zinkler M (2008). Früher entlassen – schneller im Maßregelvollzug? Zum Verhältnis von allgemeiner und forensischer Psychiatrie. Recht Psychiatr.

[11] Fazel S, Danesh J (2002). Serious mental disorder in 23 000 prisoners: a systematic review of 62 surveys. lancet.

[12] Fazel S (2016). Mental health of prisoners: prevalence, adverse outcomes, and interventions. Lancet Psychiatry.

[13] Von Schönfeld CE (2006). Prävalenz psychischer Störungen, Psychopathologie und Behandlungsbedarf bei weiblichen und männlichen Gefangenen. Nervenarzt.

[14] Schildbach S, Schildbach C (2018). Criminalization through transinstitutionalization: a critical review of the Penrose hypothesis in the context of compensation imprisonment. Front Psychiatry.

[15] Forschungsnetzwerk Wohnungslosigkeit und Gesundheit an der Charité (2020) Zählung wohnungsloser Menschen in psychiatrischen Krankenhäusern. https://wohnungslosigkeit-gesundheit.charite.de/forschung/projekte/zaehlung_wohnungsloser_menschen_in_psychiatrischen_krankenhaeusern/. Zugegriffen: 19. Apr. 2020

[16] Statistisches Bundesamt (2019) Rechtspflege: Strafvollzug – Demographische und kriminologische Merkmale der Strafgefangenen zum Stichtag 31.3. Fachserie 10, Reihe 4.1

[17] Statistisches Bundesamt (2020) In der Strafverfolgungsstatistik erfasste Personen mit Untersuchungshaft. https://www.destatis.de/DE/Themen/Staat/Justiz-Rechtspflege/Tabellen/erfasste-personen-uhaft.html. Zugegriffen: 19. Apr. 2020

[18] Flammer E, Steinert T (2015). Involuntary medication, seclusion, and restraint in German psychiatric hospitals after the adoption of legislation in 2013. Front Psychiatry.

[19] Flammer E, Steinert T (2015). Auswirkungen der vorübergehend fehlenden Rechtsgrundlage für Zwangsbehandlungen auf die Häufigkeit aggressiver Vorfälle und freiheitseinschränkender mechanischer Zwangsmaßnahmen bei Patienten mit psychotischen Störungen. Psychiat Prax.

[20] Lehmann M (2019) Erfahrungen eines substituierenden Anstaltsarztes. Vortrag auf dem Symposium „Substitutionsbehandlung Opiatabhängiger in Haftanstalten“ auf dem DGPPN Kongress 2019

[21] Konrad N (1997). Psychiatrie im Justizvollzug. Recht Psychiatr.

[22] Statistisches Bundesamt (2015) Strafvollzugsstatistik: Im psychiatrischen Krankenhaus und in der Entziehungsanstalt aufgrund strafrichterlicher Anordnung Untergebrachte (Maßregelvollzug) – 2013/2014. Wiesbaden

[23] Konrad N, Missoni L (2001). Psychiatrische Behandlung von Gefangenen in allgemeinpsychiatrischen Einrichtungen am Beispiel von Nordrhein-Westfalen und Rheinland-Pfalz. Psychiatr Prax.

[24] Konrad N (2000). Psychisch Kranke im Justizvollzug-Sicht des forensischen Psychiaters. Z Arztl Fortbild Qualitatssich.

[25] Knecht, G (2020) Vortrag am 30. Jan. 2020 in der Asklepios Klink Nord-Ochsenzoll, Hamburg

[26] Fuller Torrey E (2015). Deinstitutionalization and the rise of violence. CNS Spectr.

